# Field-programmable beam reconfiguring based on digitally-controlled coding metasurface

**DOI:** 10.1038/srep20663

**Published:** 2016-02-10

**Authors:** Xiang Wan, Mei Qing Qi, Tian Yi Chen, Tie Jun Cui

**Affiliations:** 1 State Key Laboratory of Millimeter Waves, School of Information Science and Engineering Southeast University, Nanjing 210096, China

## Abstract

Digital phase shifters have been applied in traditional phased array antennas to realize beam steering. However, the phase shifter deals with the phase of the induced current; hence, it has to be in the path of each element of the antenna array, making the phased array antennas very expensive. Metamaterials and/or metasurfaces enable the direct modulation of electromagnetic waves by designing subwavelength structures, which opens a new way to control the beam scanning. Here, we present a direct digital mechanism to control the scattered electromagnetic waves using coding metasurface, in which each unit cell loads a pin diode to produce binary coding states of “1” and “0”. Through data lines, the instant communications are established between the coding metasurface and the internal memory of field-programmable gate arrays (FPGA). Thus, we realize the digital modulation of electromagnetic waves, from which we present the field-programmable reflective antenna with good measurement performance. The proposed mechanism and functional device have great application potential in new-concept radar and communication systems.

Compared with three-dimensional (3D) bulk metamaterials, planar metasurfaces are capable of manipulating electromagnetic waves within more compact space^1^,^2^,^3^,^4^,^5^, thus leading to smaller volume and easier fabrication. Although both bulk metamaterials and metasurfaces are composed of artificial structures, their physical natures are different due to the fact that the field averaging on a surface cannot be accurately described by the effective permittivity and permeability, which are essentially 3D constitutive parameters. The surface electric and magnetic susceptibilities are firstly proposed to characterize the metasurfaces^6^,^7^, and these parameters are manipulated purposely to cancel the scattered waves in order to produce ultrathin mantle cloaks^8^,^9^,^10^. The generalized Snell’s law^11^, which explains the phenomenon of anomalous refractions and reflections, gives birth to lots of unprecedented metasurface lenses or plates by designing abrupt phase variations^12^,^13^,^14^,^15^. The spin-orbit interaction^16^ and optical angular momentum^17^ have been invested by utilizing different kinds of metasurfaces. The polarization, as another important characteristic parameter of electromagnetic waves, has also been manipulated by metasurfaces, such as polarization converters^18^,^19^,^20^ and multi-functional devices using different polarizations^21^,^22^,^23^. In addition, controls of amplitudes have been reported by designing both the geometrical configuration and angular orientation of each unit of the metasurfaces^24^. More complicated modulations of the electromagnetic waves have been realized by using system-level design tools, such as transformation optics^25^,^26^ and holographic technology^27^,^28^.

Most of the mentioned modulations are based on the gradiently varied unit cells to approximate the pre-designed successive parameter distributions, and hence are considered as analog modulations. Although digital modulations have been applied in processing communication signals (i.e., currents), and spatial light modulators have already been used to modulate lights digitally^29^,^30^, the concept of digital modulation directly to the electromagnetic wave has not been introduced into the community of metamaterials until the recent publication^31^. Cui *et al.* proposed the functional designs by introducing coding metasurfaces, in which the binary states are represented by two different values of reflective phases. Overall, the coding bits can be binary phases, binary amplitudes, or even binary polarizations.

In this work, we present a field-programmable reflective array antenna which consists of a horn antenna and a reflective coding metasurface. The binary-phase element and chessboard configuration scheme are adopted to construct the coding metasurface. By loading a pin diode in each element, the binary codes of the metasurface are controlled by field-programmable gate arrays (FPGA) directly. Therefore, the main lobes of the scattered fields from the coding metasurface are steerable at the same frequency by varying the lattice size of the chessboard configuration. Simulation results and experimental measurements validate the new-type beam steering antenna without using many phase shifters.

## Theoretical methods and designs

**Figure 1:**
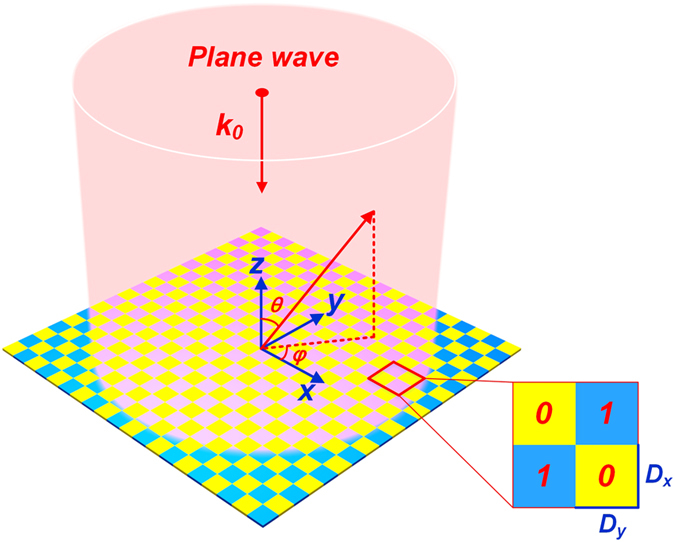
A coding metasurface illuminated by plane electromagnetic waves. The inset shows a sub-array consisting of four lattices, in which the blue one represents the state “1”, and the yellow one represents the state “0”. Each lattice has the dimension of *D*_*x*_ × *D*_*y*_.

Figure 1 illustrates a concept diagram of the coding metasurface which is constructed by periodically arranging the sub-array shown in the inset, in which the blue and yellow lattices represent the states of “1” and “0”, respectively. For each lattice, the scattered electric field intensity in the far-field region can be expressed as


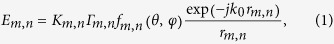


in which, 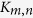 is the scale coefficient, 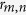 is the distance between the lattice and the observation point, 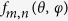 is the scattering pattern function of each lattice, and 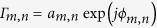, where 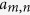 and 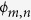 are the amplitude and phase of the reflection coefficient, respectively.

For coding metasurfaces, it is usually safe to omit the effect of 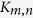 and 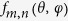 when calculating the array pattern function, because the metasurface units are much smaller than the wavelength and the detailed information of the unit is vague in the far-field region. If only the phase differences between the coding lattices “1” and “0” are considered, then the pattern function of the whole coding metasurface, when illuminated by plane waves, can be written as


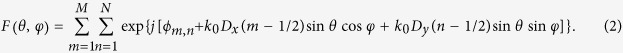


Observing the equation, one can find that the key factor of separating the double summation is to find an adequate distribution of the reflective phase 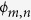. The consistent value of 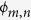 ensures the separation of the double summation but leads to mirror reflection and immovable radiation beam. The chessboard configuration of 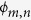 may be the simplest nontrivial scheme to separate the double summation. Supposing that 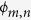 of coding lattice “1” has the value of *π*, and 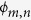 of coding lattice “0” has the value of 0, Eq. (2) is then rewritten as


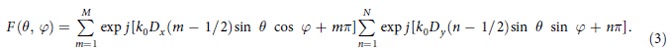


After calculating the summation, the amplitude of the pattern function is deduced as


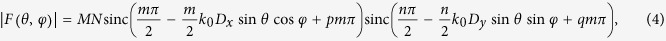


in which 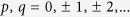. It is clear that, for the first diffraction orders, the maximum value of 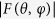 appears at the direction of


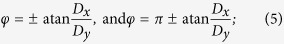



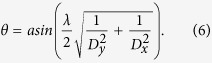


These expressions indicate the possibility to realize the beam sweeping at the same frequency by changing the lattice size. For the coding metasurface, it means changing the distribution of the digital codes.

**Figure 2:**
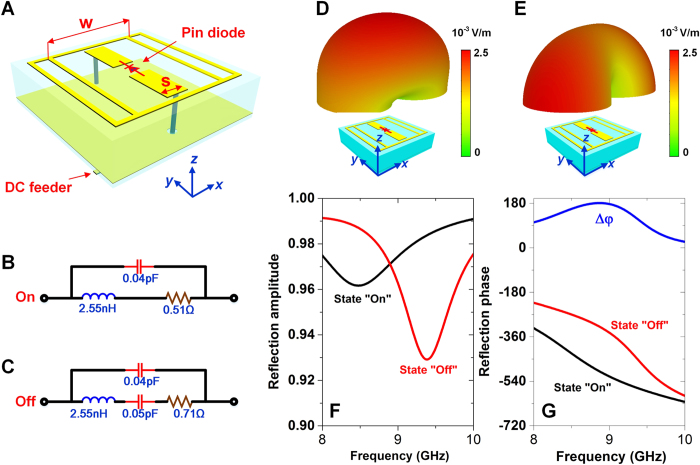
Properties of the binary unit. **(A)** The detailed structure of the binary unit which consists of three layers. Here, S = 1 mm, W = 4.3 mm, the height between the top and middle layers is 1.6 mm, while the height between the middle and bottom layers is 0.2 mm. The periodicity of the unit is 7 mm and the size of the outer copper square is 6 mm. All line widths are 0.2 mm. **(B,C)** The effective circuit models of the pin diode at the state “on” and “off”, respectively. **(D,E)** The scattered electric-field patterns of the binary unit at the states “on” and “off”, respectively. **(F)** The reflection amplitudes of the binary unit. **(G)** The reflection phases of the binary unit.

To construct the coding metasuface, a binary unit is firstly proposed. Figure 2(A) illustrates the structure of the unit cell, in which a pin diode (SMP1320 from SKYWORKS) is loaded. A direct-current (DC) feeding line is introduced to switch the states of pin diode. Commercial software, the CST Microwave Studio, is used to analyze the properties of the coding unit. In numerical simulations, the “on” and “off” states of the pin diode are represented by effective circuits displayed in Fig. 2(B,C). The field patterns of the binary unit at the states “on” and “off” are illustrated in Fig. 2(D,E), respectively. The uniformity in region near to the normal axis is better than that in other regions, indicating that the pattern function of the binary unit barely affect the field patterns of the whole coding metasurface in region with small elevation angle. With the specified structural parameters, the binary unit shows dispersive reflection characteristics as shown in Fig. 2(F,G). We observe that the perfect binary state occurs at 8.9 GHz, where the states “on” and “off” possess identical reflective amplitude and opposite reflective phases. These working states satisfy the assumptions in Eq. (2), and hence the presented units can be used to produce the programmable coding metasurface.

**Figure 3:**
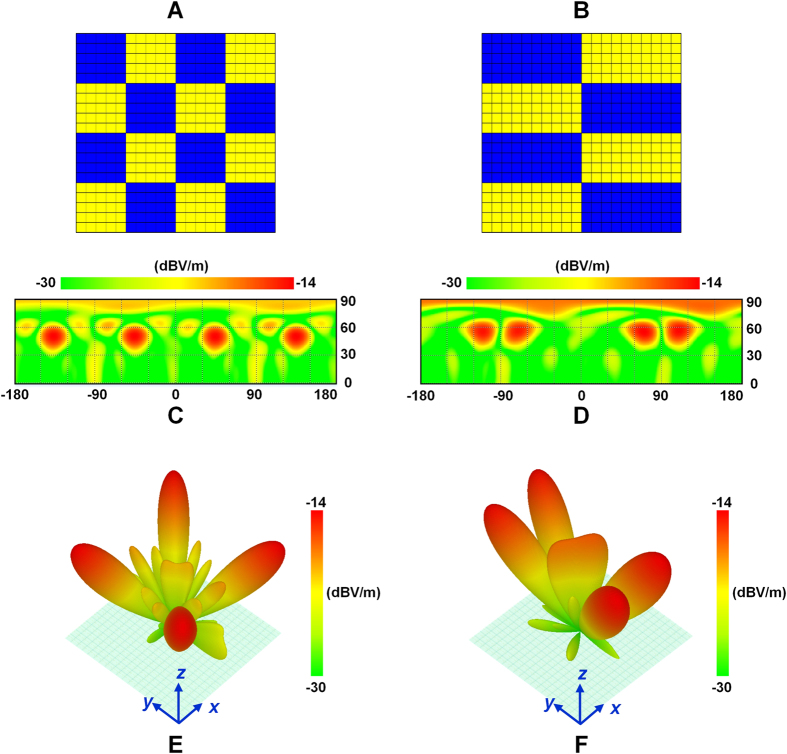
The scattered electric field patterns of the coding metasufaces with different code configurations when illuminated by plane waves. **(A)** The chessboard configuration with the lattice size of 5 × 5 units. **(C,E)** 2D and 3D views of the scattered field patterns corresponding to the configuration in **(A)**. **(B)** The chessboard configuration with the lattice size of 10 × 5 units. **(D, F)** 2D and 3D views of the scattered field patterns corresponding to the configuration in **(B)**.

The designed coding metasurface is comprised of 400 (20 × 20) binary units. Figure 3(A) shows a standard chessboard configuration, in which each lattice consists of 25 (5 × 5) binary units. Here, the blue lattices represent codes “1”, corresponding to the “off” states of the binary units; while the yellow lattices represent codes “0”, corresponding to the “on” states of the binary units. When the binary metasurface shown in Fig. 3(A) is vertically impinged by plane waves, according to Eq. (5,6), the main lobes of scattered waves appear at the directions of (43°, 45°), (43°, 135°), (43°, −135°), and (43°, −45°), which are verified by the full-wave simulations presented in Fig. 3(C,E). Note that the values of vertical axes in Fig. 3(C,D) are 90°−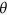. Changing the lattice size leads to transitional directions of the main lobes. In Fig. 3(B), the lattice consists of 50 (10 × 5) binary units, implying that the main lobes are directing to (32.6°, 63.4°), (32.6°, 116.6°), (32.6°, −116.6°), and (32.6°, −63.4°), respectively, which are also verified by Fig. 3(E,F).

**Figure 4:**
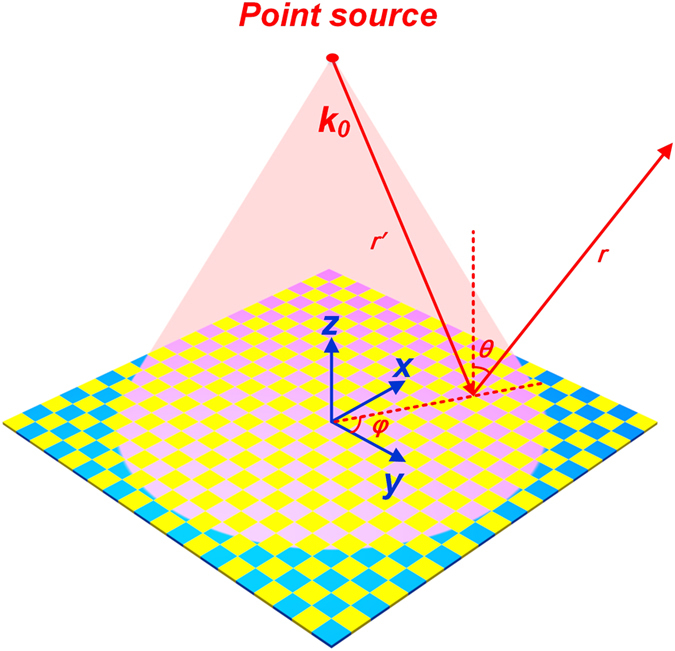
The coding metasurface illuminated by a point source.

The proposed coding metasurface is a reflected surface, and hence we have to design a source before it is used as an antenna. The plane waves used in the analyses are practically difficult to achieve in the near-field region. Even though they are achieved, the aperture of the source may be comparable with the size of coding metasuface, thus leading to huge shielding effect. If the source is non-planar wave, the phase variations on the units of the coding metasurface have to be compensated for the impinging wavefronts. Figure 4 demonstrates the schematic diagram of the coding metasurface under the illumination of a point source. In this case, the scattered electric field intensity of each lattice can be expressed as


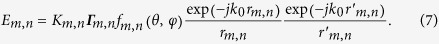


Compared with Eq. (1), this equation considers the phase effect of the source. Using the same assumption as that in Eq. (1), the pattern function of the coding metasurface is expressed as


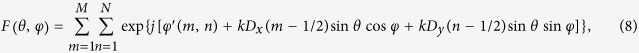


in which 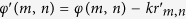. It means that, when the point source is used, the binary phase becomes 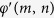, and 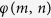 possess gradient values rather than binary values. In order to perform the phase compensation, the unit structures of the coding metasurface are no longer fixed. In other words, the phase variations of the source wavefronts are compensated by varying the geometry of unit structures. Fortunately, once the compensation is accomplished, it works for any configuration of the binary codes since the relative positions between the source and each unit structure are fixed. It only needs switching the pin diodes to change the binary codes.

By scanning the values of S and W (see Fig. 2), a database of the reflection amplitudes (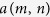) and phases (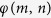) are established. For convenience, integers *I* (*I* = 0, 1, …, 9) and *J* (*J* = 1, 2, …, 31) are used to express the values of S and W. The expressions are written as


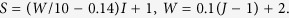


**Figure 5:**
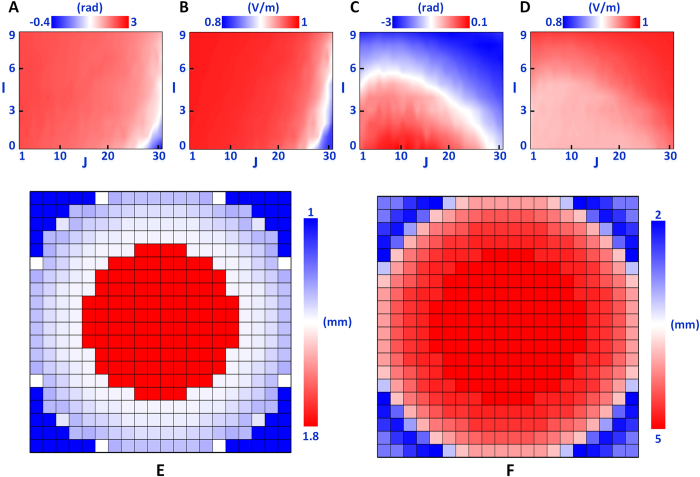
Procedures of the phase compensation. **(A,B)** The reflection amplitudes and phases of the units with different values of S and W when the pin diodes are turned off. **(C,D)** The reflection amplitudes and phases of the units with different values of S and W when the pin diodes are turned on. **(E,F)** The distributions of the optimized values of S and W, respectively.

Figure 5(A,B) demonstrate the reflection amplitudes and phases for different geometries when the pin diode is switched off (code “1”), while Fig. 5(C,D) show the cases when the pin diode is switched on (code “0”). With the aid of the least-square algorithm, the best values of S and W for each unit structure of the metasurface are achieved. Figure 5(E,F) display the value distributions of S and W, respectively. At last, the unit of the coding metasurface compensates the phase variations of the impinging wavefronts, meanwhile, maintains the opposite phase values for codes “1” and “0”.

**Figure 6:**
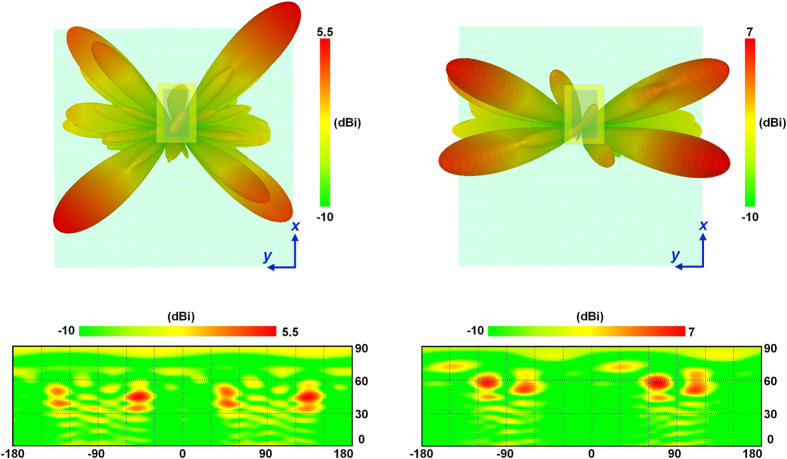
The scattered field patterns of the coding metasurfaces with different code configurations when illuminated by the point source. **(A,C)** 3D and 2D views of the scattered field patterns corresponding to the configuration in Fig. 3(A). (**B,D)** 3D and 2D views of the scattered field patterns corresponding to the configuration in Fig. 3(B).

In full-wave simulations, a rectangular horn antenna is used as the source. The distance between the source and metasurface is chosen as 250 mm so that the biggest incidence angle of the metasuface units is not larger than 20°. Hence the database established by normally illuminating the units is still usable. Figure 6(A,C) present the simulated field patterns when the coding metasurface obeys the code distribution in Fig. 3(A), while the field patterns corresponding to the code distribution in Fig. 3(B) are presented in Fig. 6(B,D). Except for some divergences which should be resulted from the non-uniformity of the field intensity on the metasurface, the main lobes appear around the predicted directions.

## Experiments and discussions

**Figure 7:**
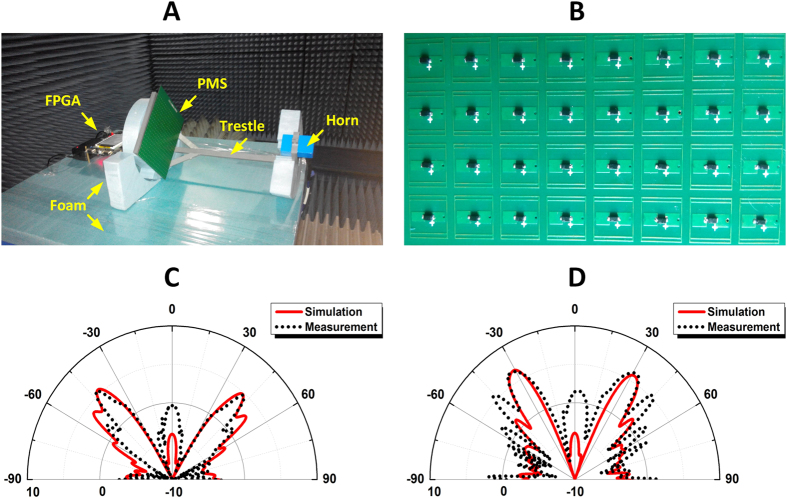
Experiments and measured results. **(A)** The experimental setup. **(B)** A portion of the programmable coding metasurface. **(C)** The comparison of the simulated and measured gains when the azimuth angle is 63.4°. (**D**) The comparison of the simulated and measured gains when the azimuth angle is 45°.

An experiment model of the field-programmable reflective antenna is displayed in Fig. 7(A), in which the source and coding metasurface are installed at a trestle. The field-programmable gates array (FPGA) is used to control the digital states of each lattice (5 × 5 units). Figure 7(B) shows a portion of the coding metasurface. The pre-designed distributions of binary codes have been written into the internal memory of FPGA. Through the real-time communications between the coding metasurface and the internal memory, the binary codes on the metasurface are programmable. As demonstrations, the codes in Fig. 3(A,B) are written into the internal memory of the experiment model, which is then measured in a microwave chamber. Figure 7(C,D) compare the simulated and measured gains for the two kinds of code distributions, from which the main lobes are observed in the predicted directions, proving that the presented programmable reflective antenna is capable of steering the beams. The enlarged side lobes in measured results are mainly caused by the fact that the units of the designed metasurface are not placed with exact periodic boundaries as what we have considered in designing a single unit. Furthermore, the packages and soldering processes of the pin diodes may introduce errors into the effective circuit models.

Theoretically, arbitrarily steering directions of the main lobes can be realized by adjusting the lattice size. However, Eq. (6) has indicated that the lattice size possesses a lower limit when considering the value range of sinusoidal functions. There comes a problem that the marginal lattices may not be integrated ones since the fabricated metasurface has limited size, and the problem gets worse for the lattices with large size which corresponds to large elevation angle. Hence, it can be predicted that the proposed antenna performs better when the main lobes are not very close to the normal direction. On the other hand, the relatively small lattice size guaranties the uniform reflection phase and amplitude of each lattice when the metasurface is illuminated by a point source. This explains why the measured results for the codes in Fig. 3(A) are better than those of codes in Fig. 3(B).

## Conclusion

We have presented a field-programmable reflective antenna based on the coding metasurface in the microwave frequency, in which the binary units are realized by loading pin diodes to subwavelength artificial structures. By switching the pin diodes, the binary units possess opposite phases, which are represented by codes “1” and “0”. Then FPGA is used to configure the code distributions on the coding metasurface, thus making the main lobes of the scattered waves digitally reconfigurable at the same frequency. This antenna is different from typical beam steering antenna since the concomitant beams cannot be eliminated. As a result, the antenna cannot resolving the targets which are detected by grating lobes simultaneously. However, the grating lobes still have positive effects. For an example, if priori knowledge about rough directions of targets are obtained, the ambiguity of the antenna can be eliminated, then we can rapidly position the targets. Take a step back, if there are not priori knowledge, this antenna can rapidly provide four possible directions of the targets, which can be priori knowledge of the subsequent detections. This technique can possibly reduce the position times and the complexities of the follow-up detection radar.

More recently, the coding metasurfaces have been presented in the terahertz frequencies by introducing fractal Minkowski particles^32^ and circular-ring resonator^33^. Hence it is possible to realize the real-time controllable digital beam steering in the terahertz regime using the proposed method in the future.

## Additional Information

**How to cite this article**: Wan, X. *et al.* Field-programmable beam reconfiguring based on digitally-controlled coding metasurface. *Sci. Rep.* **6**, 20663; doi: 10.1038/srep20663 (2016).
